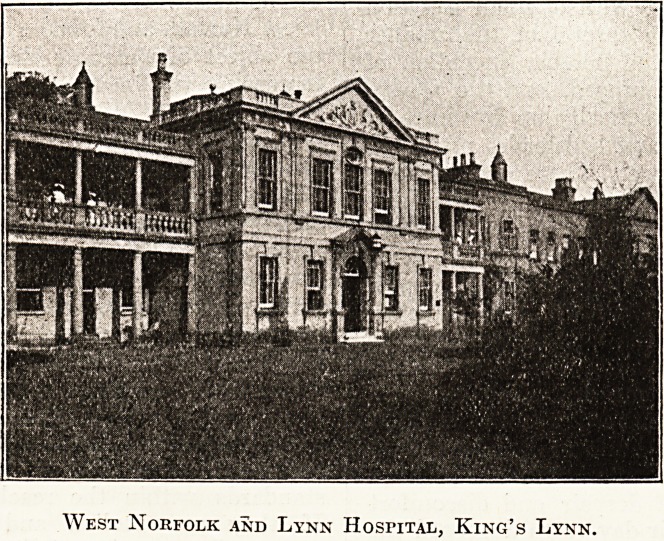# Reports on Hospitals of the United Kingdom

**Published:** 1914-01-10

**Authors:** Henry Burdett


### REPORTS ON

### Hospitals of the United Kingdom.

By SIR HENRY BURDETT, K.C.B., K.C.V.O.

SERIES III.

### THE WEST NORFOLK AND LYNN HOSPITAL.

This hospital, established in 1834, was partially reconstructed in 1912 as a memorial to King Edward VII. Previous to its reconstruction, this hospital must have been in a most lamentable condition. In 1912 the new lavatories, bathrooms, and ward kitchens were completed. It would have been wisdom on the part of the managers to have made a supreme effort then to raise a building fund of sufficient amount to enable the hospital to be rebuilt. This might then have been done. Some of the Committee, we believe, wished this course to be adopted, but they were, unfortunately, out-voted.

West Norfolk and Lynn Hospital, King's Lynn.

### The Committee's Task.

As matters stand at present the task of the Committee is to obtain sufficient funds, say £2,000, to provide accommodation for the nursing staff and to equip fully the wards and other portions of this hospital, which are, in some respects, shabby to desolation. They have, further, to raise an additional £600 to £1,000 a year in revenue. This should not be a very difficult task if they are wise enough to enlarge the present area of givers, by adopting new means of raising income in small sums. This can be done by enlisting the help of the women of all classes everywhere throughout the district, who can successfully organise a crusade of personal service to extend the privilege to each citizen to give of themselves in the days of health in the cause of the sick. We had the pleasure of meeting the honorary financial secretary, Mr. C. Bristow, of Barclay's Bank, who has the best interests of the hospital at heart. He would, we are confident, prove an able leader in the new crusade to help the hospital through personal service, and thereby immeasurably to add to the individual happiness of a great many people of all classes resident in this part of the county of Norfolk.

### The Right Spirit, but Many Laggards.

In what we have just said, and in what follows, we are fully sensible of the efforts that have been made by house-to-house collections in the villages, through the "Forward Association," by means of linen and mending guilds, and in other ways, to help the hospital. All these movements show the right spirit. But the accounts exhibit that there is a large section of the community which must take little or no interest in the work of maintaining the West Norfolk and Lynn Hospital. Thus the whole amount received from the many chapels and places of worship, including the Free Churches, is at present less than £30 a year. Surely, having regard to the number of patients who belong to the Free Churches in this community which the hospital benefits, it is time that the pastors and leaders of these Churches should bestir themselves to ensure that the gifts from their congregations shall become more worthy of their numbers and importance. We say nothing of the debt which the Free Churches owe to the hospital for the benefits they have so generally received through its instrumentality.

### Inside the Hospital.

The most attractive feature of the hospital is the outside, which, though apparently erected at diverse times and for diverse purposes, presents, on the whole, an imposing frontage which is homely rather than stately. A walk through the hospital reveals the somewhat ramshackle character of the ground floor, most of which is taken up by the accommodation for out-patients, the casualty department, a dental room, and the children's ward at the end. The most workmanlike feature on the ground floor is undoubtedly the dental room, which had the appearance of business, and yet we were informed that the dentist only attends on Saturdays! On the ground floor to the left of the entrance are the matron's apartments, those for the house surgeon, rooms for some of the nursing staff, and the kitchens, pantry, and what is called the washhouse, where apparently septic linen is dealt with. When we visited it, it was being used as a carpenter's shop.

### Forbidding, Dull, and Miserable!

The principal wards are on the first floor, and are divided about equally between men and women, there being 18 beds in the male wards and 20 beds in the female wards, with a small isolation room. This hospital, like several of the smaller Norfolk hospitals, with the notable exception of Cromer, is indifferently equipped, and the wards, containing, as they do, practically no furniture or comforts except the beds and bedsteads, presented a rather desolate appearance. There are, besides, two passage ways, devoted respectively to men and women, which are called day rooms. When we entered the male day room we found it the most forbidding, dull, and miserable place we have seen for a long time. Its furniture was old, decrepit, contemptible, and had all the worst aspects of the worst kind of accommodation the worst workhouse in the country can contain. The women's day room, though the furniture was not what it ought to be, did not look quite so bad, but then it did not contain any patients. We are inclined to think that the look of despair and discomfort on the men's faces in their day room deepened the impression of cruelty which this apartment gave to the visitor. Are there not enough men and women among the 20,000 inhabitants of King's Lynn, and many additional thousands of inhabitants in West Norfolk, which latter district supplies about half the patients received by this hospital each year, with enough intelligence and enough heart to be cut to the quick, when they realise the responsibility which rests upon them for allowing the patients when out of bed to have no resting place but these passage ways of discomfort and gloom? Fancy the sad position of sick people who, at the moment when they are just able to leave their beds after a severe illness, when they imperatively require the stimulus of cheerfulness and restful surroundings to complete their cure, have no day room or comforts whatever. Cheerfulness in the sick is the high road to recovery, but this high road is closed at present to the in-patients of this hospital for the want of a few pounds and a little driving force, which would sweep out with a rush the apology for furniture at present constituting the only equipment of these mis-called day rooms. He would be indeed a worthy citizen who would take out all this rubbish from the hospital and make a bonfire of it, whilst the workmen from a neighbouring emporium were replacing it by comfortable chairs and new pictures, a couple of couches, and presentable tables, with a few games to amuse the patients who are sufficiently convalescent. We expect, of course, the answer will be that the hospital has no money to spend. Our reply is, "In the name of goodness what are the men and women who represent the most prosperous, and therefore the most responsible class, including the clergy of all denominations, doing, that they do not turn their attention at once to the equipment of this hospital and provide that what is necessary shall be done in the course of the next few weeks?" Where there is a will there is a way, and the sooner that way is found by the people of King's Lynn the better will it be for the reputation of this ancient seaport.

### Wanted! A Ladies' Association.

Will not Mrs. Halcombe Ingleby place herself at the head of the women of King's Lynn and West Norfolk and found a Ladies' Association with the object of widening the area of givers to, and increasing the income of this hospital by £500 a year at least? What the hospital suffers from is a failure, on the part of the Committee and of the people whom it serves, to recognise that the treatment at the hospital, so far as the medical staff is concerned, is modern and up to date, and that this great advance in treatment is necessarily some 25 per cent. more costly, but is productive of infinitely better results as regards the welfare of the patients than were possible of attainment thirty years ago. We have often said that a city has the hospital which its citizens deserve. If the people of King's Lynn and West Norfolk are wise they will gladly supply the extra £500 or £600 a year to keep the treatment at this hospital on a level with the highest standards within the reach of its medical staff. If they are foolish and wilfully blind, they must suffer a serious falling off in the reputation and efficiency of the hospital, and cause a material diminution in the earning power of those householders and their dependants who have as their chief strength in illness the King's Lynn and West Norfolk Hospital.

**Some Cottage Hospitals in Norfolk and Suffolk will be reported on next week.**

## Figures and Tables

**Figure f1:**